# Behavioral Lateralization and Optimal Route Choice in Flying Budgerigars

**DOI:** 10.1371/journal.pcbi.1003473

**Published:** 2014-03-06

**Authors:** Partha S. Bhagavatula, Charles Claudianos, Michael R. Ibbotson, Mandyam V. Srinivasan

**Affiliations:** 1 ARC Centre of Excellence in Vision Science, Australian National University, Acton, Canberra, Australian Capital Territory, Australia; 2 Research School of Biology, Australian National University, Acton, Canberra, Australian Capital Territory, Australia; 3 Queensland Brain Institute, The University of Queensland, St Lucia, Brisbane, Queensland, Australia; 4 National Vision Research Institute, Australian College of Optometry, Carlton, Melbourne, Victoria, Australia; 5 School of Information Technology and Electrical Engineering, The University of Queensland, St Lucia, Brisbane, Queensland, Australia; Northeastern University, United States of America

## Abstract

Birds flying through a cluttered environment require the ability to choose routes that will take them through the environment safely and quickly. We have investigated some of the strategies by which they achieve this. We trained budgerigars to fly through a tunnel in which they encountered a barrier that offered two passages, positioned side by side, at the halfway point. When one of the passages was substantially wider than the other, the birds tended to fly through the wider passage to continue their transit to the end of the tunnel, regardless of whether this passage was on the right or the left. Evidently, the birds were selecting the safest and quickest route. However, when the two passages were of equal or nearly equal width, some individuals consistently preferred the left-hand passage, while others consistently preferred the passage on the right. Thus, the birds displayed idiosyncratic biases when choosing between alternative routes. Surprisingly - and unlike most of the instances in which behavioral lateralization has previously been discovered - the bias was found to vary from individual to individual, in its direction as well as its magnitude. This is very different from handedness in humans, where the majority of humans are right-handed, giving rise to a so-called ‘population’ bias. Our experimental results and mathematical model of this behavior suggest that individually varying lateralization, working in concert with a tendency to choose the wider aperture, can expedite the passage of a flock of birds through a cluttered environment.

## Author Summary

Birds display a clear mastery of the skill of flying rapidly and safely through complex and cluttered environments. An example of this can be viewed at http://www.youtube.com/watch?v=p-_RHRAzUHM, which shows a bird flying at high speed through a dense forest. Such mastery requires the ability to determine, from moment to moment, which of several possible routes would provide the safest and quickest passage. Our study is one of the first to investigate how birds achieve this. Our experiments reveal that, when flying budgerigars are required to choose between two passages, they tend to favor the wider passage. However, this tendency is superimposed upon a bias that, surprisingly, varies from bird to bird: some individuals show an intrinsic preference for the left-hand passage, and others for the passage on the right. This is very different from handedness in humans, where the majority of humans are right-handed. We develop a mathematical model of the interaction between the birds' individual biases with their tendency to prefer the wider passage. The model reveals that this interplay is actually beneficial – it can expedite the passage of a flock of birds through a complex environment.

## Introduction

A bird flying through a complex and cluttered environment relies heavily on the use of visual cues to rapidly choose between alternative routes and avoid collisions with intervening obstacles. Goshawks, for example, display an impressive ability to fly through dense environments at high speeds [1]. Neural correlates of obstacle detection have been investigated in pigeons, where it was shown that neurons in the nucleus rotundus of the brain respond to a visual stimulus that depicts a moving object on a collision course [2]. While such neurons probably constitute part of an “early warning” system, it remains to be seen how the responses of such neurons contribute to the planning of an efficient and safe flight trajectory.

What strategies do birds adopt to fly efficiently through complex environments? Here we investigate the behavior of budgerigars when they are offered a choice between two passages, presented side by side, through which they can fly. The relative widths of the passages are varied to investigate the rules that govern the birds' choices, and to examine whether these rules can facilitate rapid flight of a flock of birds through dense environments.

## Results

**Figure 1:**
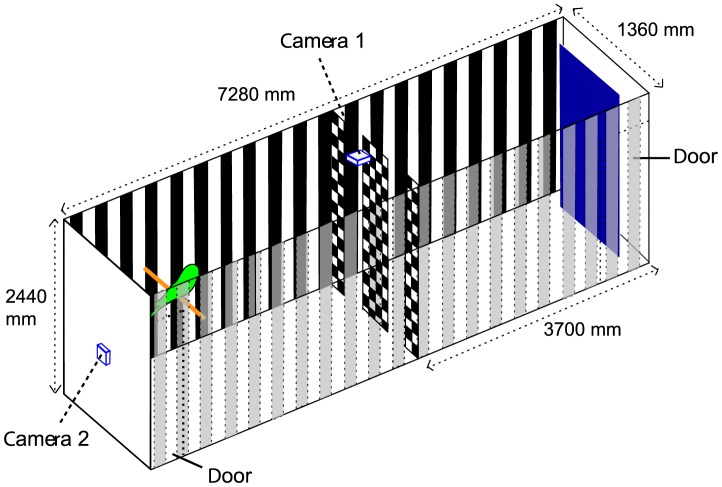
Experimental configuration. Details in text.

We flew birds individually in a tunnel that presented a barrier with two apertures, positioned side by side halfway along its length, as illustrated in Fig. 1. The frequency with which the birds chose one aperture or the other was recorded, as the relative sizes of the two apertures were varied.

**Figure 2:**
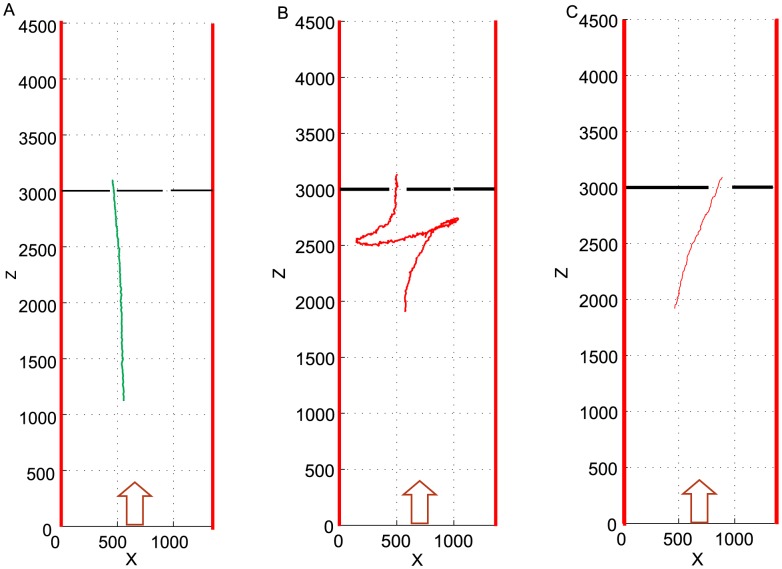
Examples of birds choosing between two apertures. The red arrow denotes the direction of bird flight. The widths of the left- and right-hand apertures are respectively 60 mm and 40 mm in (A), 90 mm and 10 mm in (B), and 0 mm and 100 mm in (C).

Some of the flights were filmed and reconstructed in 3D using high-speed stereo cameras, as described in “Methods”. Three examples of such flights, as viewed from above, are shown in Fig. 2. In Fig. 2*A* a bird selects the left-hand aperture (of width 60 mm) over the right-hand aperture (of width 40 mm). In Fig. 2*B* a bird initially flies toward the right-hand aperture (of width 10 mm), but possibly finds it too narrow and then chooses the left-hand aperture (of width 90 mm). In Fig. 2*C* a bird chooses the aperture on the right (of width 10 mm) because the left-hand aperture has zero width (i.e. is non-existent). [Birds can, and do occasionally choose to fly through very narrow apertures because the flanking panels, made of cloth, are compliant. The wings are then folded back to allow the bird to ‘projectile’ through the slit (unpublished observations)].

A preliminary analysis of the birds' choices revealed the following general characteristics. When the apertures were very different in width, the birds tended to prefer the wider aperture, regardless of whether it was on the right side or the left. The birds appeared to be selecting the safest and quickest route. However, when the apertures were of equal (or nearly equal) width, some individuals consistently preferred the left-hand aperture, while others consistently preferred the right. Left-biased birds preferred the left-hand aperture, while right-biased birds preferred the aperture on the right. Thus – as we shall demonstrate in greater detail below – each bird had its own, characteristic, side bias.

What are the factors that govern the choice of aperture? We examined this question in greater detail by investigating how the bird's choices changed as the relative sizes of the two apertures were varied systematically. This was done by varying the position of the central panel that separated them, as described in the ‘Methods’ section. As the central panel was moved from its extreme left-hand position to its extreme right-hand position (in steps of 10 mm), the width of the left-hand aperture increased progressively from 0 mm to 100 mm, and the width of the right-hand aperture decreased progressively from 100 mm to 0 mm, as shown in Table 1.

**Table 1:** Widths of left-hand and right-hand apertures.

Aperture widths		
Experimental condition	Left	Right
1	0 mm	100 mm
2	10 mm	90 mm
3	20 mm	80 mm
4	30 mm	70 mm
5	40 mm	60 mm
6	50 mm	50 mm
7	60 mm	40 mm
8	70 mm	30 mm
9	80 mm	20 mm
10	90 mm	10 mm
11	100 mm	0 mm

**Figure 3:**
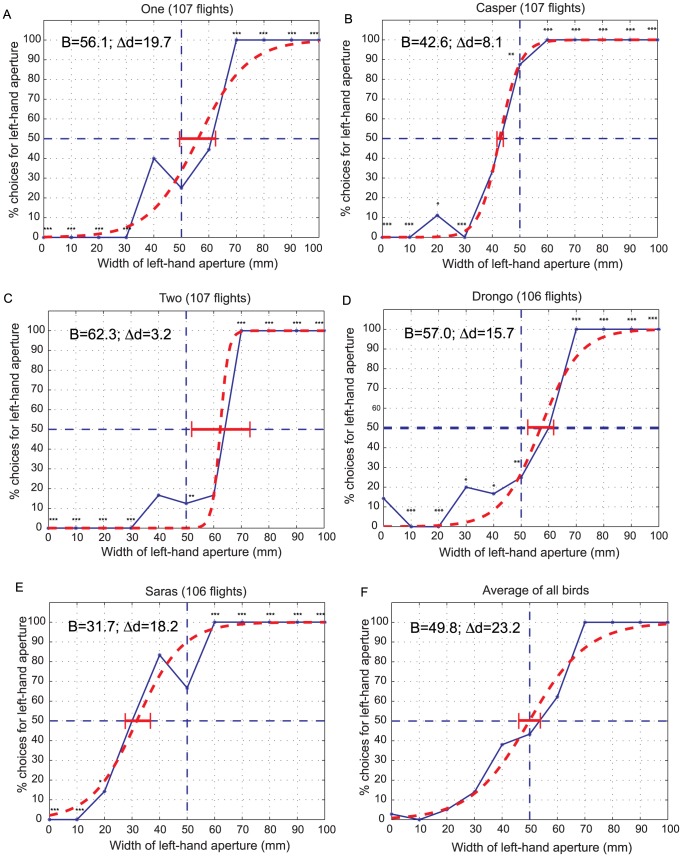
(A–E) Aperture choice profiles for birds *One, Casper, Two, Drongo* and *Saras*, showing choice frequencies for the left-hand aperture as a function of its width. The dashed vertical line represents the condition when both apertures are of equal width (50 mm). The dashed horizontal line represents the random-choice level of 50%. The symbols next to each data point indicate a statistically significant difference of the choice frequency from the random-choice level of 50%, calculated as described in ‘Methods’. [p<0.05: (*); p<0.02: (**) and p<0.00001: (***)]. The red dashed curve in each panel displays a fit of the data to a logistic function, as described in the Supporting Information. (**F**) Average preference for the left-hand aperture as a function of its width, obtained by pooling the aperture choice profiles of all 5 birds (Fig. 3A–E). The fitted values of the parameters B and Δd of the logistic function are shown in each panel. The horizontal red error bar in each panel represents the 95% confidence interval for the estimated value of B.

Fig. 3*A* shows how the choice frequency for the left-hand aperture varied with its width, for one particular bird (bird *One*). When the two apertures were equally wide (or nearly so), the bird displayed a preference for the right-hand aperture, choosing it with a frequency of 74%. However, as the central panel was moved towards the right, making the right-hand aperture narrower than the left-hand one, the bird exhibited an increased preference for the left-hand aperture, eventually choosing it with 100% probability for left-hand aperture widths of 70 mm or greater. Conversely, when the central panel was shifted progressively towards the left, the bird showed a decreasing preference for the left-hand aperture, eventually not choosing it at all when it was 20 mm or narrower. Overall, the data suggest that bird *One* has a weak preference for the right-hand aperture, and that this bias is superimposed upon the bird's tendency to choose the larger of the two apertures (but see below).

The results of a similar experiment conducted with a different bird (*Casper*) are shown in Figure 3*B*. This bird was left-biased: when the apertures were of equal width, the bird showed a greater preference for the left-hand aperture, choosing it 87.5% of the time. This choice probability was significantly different from the random-choice level of 50% (p<0.02).

Figs. 3*C*–*E* show data from three additional birds: *Two*, *Drongo* and *Saras*. Bird *Two* possessed a preference for the right-hand aperture. This bird chose the left-hand aperture only 12.5% of the time when the two apertures were equally wide, and this preference was significantly lower than the random choice level of 50% (p<0.02). *Drongo* (Fig. 3*D*) was also right-biased, but less strongly so than *Two*. *Saras* (Fig. 3*E*) was strongly left-biased.

The capacity of the birds to discriminate differences in aperture width can be quantified by fitting the choice frequency data to a logistic function that describes the choice frequency *F_L_* for the left-hand aperture as
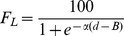
(1)where *d* is the width of the left-hand aperture. B is a bias parameter that specifies the bias of the bird, as estimated from the fitted function. It is the width of the left-hand aperture at which this aperture is chosen 50% of the time, i.e. the bird chooses randomly between the left- and right-hand apertures. If B = 50 mm, the random choice occurs when the two apertures are equally wide, and the bird is unbiased. If B<50 mm, the bird is left-biased, and if B>50 mm the bird is right-biased. α is a parameter which defines the sharpness of the bird's transition between the left-hand aperture and the right-hand one. The larger the value of α, the steeper the transition, and the sharper the discrimination of aperture width. The parameters B and α, and their confidence intervals were determined by performing a least-squares fit of the logistic function to the data for each bird and for the pooled data from all birds, as described in “Methods”. The computed values of B, and their 95% confidence intervals (Table 2) indicate that bird *One* has no significant bias, that *Casper* and *Saras* are left-biased (B<50 mm), and that *Two* and *Drongo* are right-biased (B>50 mm).

**Table 2:** Results of fit of data to a logistic function (equation (24)) with parameters B, α, and *Δd*, as described in the text and “Methods.”

Bird	B (mm)	Bias	α	*Δd* (mm)
*One*	56.1±6.5	Zero	0.11±0.07	19.7±12.6
*Casper*	42.6±1.2*	Left	0.27±0.08	8.1±2.5
*Two*	62.3±10.6*	Right	0.69±3.10	3.2±14.4
*Drongo*	57.0±4.7*	Right	0.14±0.08	15.7±9.1
*Saras*	31.7±4.7*	Left	0.12±0.06	18.2±9.1
**All birds**	49.8±4.1	Zero	0.09±0.03	23.2±11.9

The numbers show estimated values and 95% confidence limits. The asterisks identify values of B that are significantly different from 50.0, indicating a significant bias.

Table 2 also shows the increase in the width (*Δd*, in mm) of the left-hand aperture that is required for the choice frequency for the left-hand aperture to increase from 25% to 75%. This is derived from the value of α, as described in “Methods”. The smaller the value of *Δd*, the sharper the discrimination of aperture width. On this measure, bird *Two* displayed the sharpest discrimination (*Δd* = 3.2 mm) and bird *One* displayed the least sensitive discrimination (*Δd* = 19.7 mm). The mean value of *Δd*, averaged over all birds, is 13.0 mm, which indicates that, on the whole, the birds display an impressively sharp ability to discriminate aperture width.

What would be the behavior of the population as a whole? We examined this question by averaging the data from all of the birds, point by point, for each aperture width. The results are shown in Fig. 3*F*, which represents the average of the results obtained for all five birds, graphed in Fig. 3*A–E*.

The averaged curve shows no significant bias (B = 49.8; Fig. 3*F* and Table 2). This is as one might expect, given that in the group of birds that we tested, one bird had no significant bias, three birds displayed a right bias, and two others a left bias. Furthermore, while the relative preferences for the two apertures change sharply as a function of their relative widths for each individual bird (mean *Δd* = 13.0 mm), the relative preferences of the population as a whole change more gradually and smoothly (*Δd* for pooled data = 23.2 mm). The reason for this is that the sharp transition displayed by each bird occurs at a different point along the horizontal axis, because of the different biases possessed by the individual birds. This smoothing effect may have interesting implications for the behavior of a flock of birds, as we shall see in the Discussion section.

Fig. 3*F* suggests that the population, when considered as a whole, does not possess any net bias. A larger sample of birds would need to be examined before this statement can be made with complete confidence. Nevertheless, the spread of left and right biases that we have observed in the five birds that we have investigated suggests that, if there is a net bias at the level of the population (towards the left or the right), it is likely to be small.

## Discussion

In this study we have examined the way in which budgerigars approach and choose between two apertures, with a view to gaining an understanding of how birds choose routes through cluttered environments. The experiments reveal that when the birds are offered two apertures that are of equal or nearly equal width, some individuals show a preference to fly through the left-hand aperture, while others prefer to fly through the right-hand aperture. However, when the apertures are very different in width, this individual bias is overridden by a preference to choose the wider aperture, i.e. the route that is more easily traversable.

### Individual bias versus population bias

The data of Fig. 3 reveal that birds display significant lateralization in their visually guided behavior. To our knowledge, ours is the first report of lateralization in bird flight. The results reveal, furthermore, that the lateralization varies in strength and polarity from bird to bird, but has a value close to zero when averaged across several birds.

The pattern of choices that we have observed in the dual-aperture experiments is similar, in some respects, to that reported for tree swallows [3]. They found that tree swallows, when presented with two apertures of different width, tended to choose the wider aperture. However, that study did not examine how the birds' choices varied with changes in the relative widths of the two apertures – their experiments were conducted using two apertures that were either equally wide, or which differed in width by a fixed value. Furthermore, while our findings indicate a clear and strong side bias in most of the individuals that we have tested, Mandel et al. [3] state that they find no individual bias – or any net population bias – in their birds, when they chose between two equally wide apertures. However, their study presents only pooled data from all of the birds (which reveals no population bias, in agreement with our findings), but it does not provide any data or analysis of the performance of individual birds. Their study does not permit a statistical comparison of the performances of the individual birds, because each bird was tested only once in each of their experimental configurations. The question of whether tree swallows differ from budgerigars with respect to individual variations in bias, therefore, remains unresolved. However, there could well be differences between the two species, given that they tend to inhabit somewhat different environments.

So far, lateralization of vision in birds has been investigated primarily in the context of tasks that involve object detection and recognition. For example, pigeons memorize visual patterns better when they are viewed with the right eye; whilst chickens use their right eye to detect food, and their left eye to maintain a vigil against predators [4]. It has been suggested that birds that are strongly lateralized are good at multitasking. Parrots that have strongly lateralized brains are better able to use their beak as well as their feet in an experimental task that involves acquiring a food item suspended by a string [5]; Pigeons with strongly lateralized brains are better able to visually discriminate grain from grit [6].

In the above examples, the bias has been observed to occur at the population level. That is, almost all of the individuals display the same direction of bias [7]–[9]. It has been suggested that the presence of a population bias can be beneficial for species that are social: for example, a school of fish would all turn in the same direction when chased by a predator, thus ensuring that individual members do not get singled out for an attack (e.g. [10]). On the other hand, it has also been suggested that individuals of non-social (solitary) species would benefit from having individually different biases, because their escape responses would then be less predictable to a predator [7]. However, there are many documented instances of individually varying lateralization, for which the adaptive benefits – if any – remain unexplained: as in the eyeing and picking up of food by parrots [11], [12], or the use of twigs to dig out worms from holes by New Caledonian crows [13], [14].

Our study has revealed that budgerigars display individually varying lateralization when they are required to choose between to apertures. As we shall show in the following discussion and the mathematical model, this behavior can be advantageous when a flock of budgerigars attempts to fly through a densely cluttered environment.

### Benefits of individual lateralization in collective bird flight

What might be the selective advantage of having individually varying biases in the way birds use vision to guide their flight? One possibility may be an enhancement in the speed and safety with which a flock of birds can fly through dense foliage. It is clear that budgerigars confront this problem often, as do flocks of other bird species.

When a flock is faced with a choice of flying through one of two clear passages through a thicket of branches, it would be detrimental if all of the birds were to possess the same bias, say, toward the left. A population bias of this kind would make all of the birds try to fly through the left-hand passage, thus blocking each other, and slowing down as well as endangering the passage of the flock through the thicket. (The right-hand passage would not be used at all, and therefore would be wasted). On the other hand, it would also be detrimental to have no bias at all in each of the birds, because this would tend to make each individual vacillate in front of two equally wide passages before making a decision, again slowing down the progress of the flock through the thicket and increasing the likelihood of bird-to-bird collisions. Furthermore, if the two passages were of unequal size, a flock of unbiased birds would all try to fly through the wider passage, overcrowding it and again slowing down progress and increasing the likelihood of bird-to-bird collisions. The narrower passage would not attract any birds even if it were wide enough to permit safe flight, and it would thus be a ‘waste’ of a potentially useful conduit. On the other hand, if, say, half the population was left-biased and the other half right-biased, two passages of equal width would attract roughly equal numbers of birds, thus speeding up the progress of the flock through the thicket. Furthermore, the left-biased and right-biased birds would choose the left and right-hand passages without any hesitation, leading to a quicker and safer passage of the flock through the thicket. In this case, as the right-hand passage is gradually made wider than the left-hand passage, all the birds would not immediately flock to the right-hand passage: many of the left-biased birds would continue to favor the left-hand passage until it became too narrow for safe transit. Thus, a hybrid flock of left and right-biased birds would make better use of both of the available routes, and fly through the thicket more quickly.

In the next section we describe a mathematical model that characterizes the above discussion quantitatively, and demonstrates that the transit of a flock of birds through a two-passage environment will be quickest when individual birds in the flock carry different biases – ranging from an extreme left-bias, through no bias, to an extreme right-bias. We also show that the transit time of the flock would be a minimum when the members of such a flock (treated as a whole) choose each passage with a frequency that is proportional to its width. That is, the number of birds that use each passage is proportional to the width of the passage. This would ensure that both passages complete transmitting birds at the same time, and are therefore used optimally. In reality, given that the birds cannot fly through apertures that are narrower than about 30 mm, one would expect that the frequency of choosing an aperture would not increase strictly linearly with its width. Rather, it would vary as a broad sigmoidal function that has a threshold width of about 30 mm, and which attains the 100% level at a width of about 70 mm (beyond which the other aperture would be narrower than 30 mm and therefore not traversable). This is indeed the shape of the discrimination curve that is displayed by the pooled data (Fig. 3*F*).

**Figure 4:**
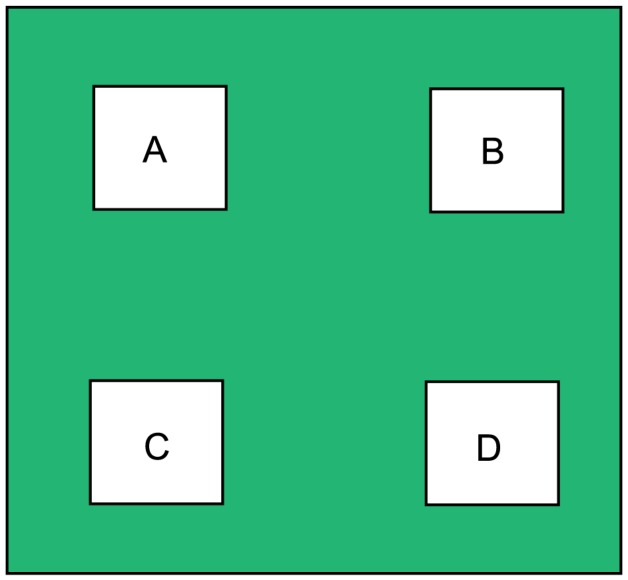
Illustration of a hypothetical example in which there are four apertures: A, B, C and D. Details in text.

The above considerations would also apply to a situation where a flock encounters several apertures. For example, let us consider a case of four equal apertures, arranged as shown in Fig. 4. Let us assume that some individuals in the flock prefer aperture A (the upper left-hand aperture), others aperture B (the upper right-hand aperture), some prefer aperture C (the lower left-hand aperture) and yet others prefer aperture D (the lower right-hand aperture). It can then be shown that this individually varying bias, acting in concert with a tendency to prefer the widest aperture when the apertures have different widths, will again lead to an optimal routing of the birds through the various apertures, and expedite the transit of the flock.

We conclude that flying budgerigars display characteristic, individual biases when choosing between alternative routes. This is the first report of lateralization in visually guided bird flight. Contrary to most other known instances of lateralization in birds, the bias occurs at the level of the individual, rather than the population. Our mathematical model of this behavior suggests that the individually varying bias, working in concert with a general tendency to prefer routes that are more easily traversable, can expedite the passage of a flock of birds through dense foliage.

Our model – which is a simplified, first attempt to characterize choices between navigable apertures – assumes that when the birds make these choices whilst in a flock, they behave independently of each other. That is, our analysis neglects any interactions that might occur among the birds when they are making their choices. Such interactions, if extant, would be an important subject of future experimentation and theory.

We note that the behavioral task that we have studied here is fundamentally different in nature from that of flocking or schooling, where interactions among individuals facilitate the coordinated movement of a group of individuals in free space (e.g. [15]–[17]). There the task is not necessarily to select the best aperture through which to fly, because all of the birds are flying at a more-or-less constant speed. Rather, each bird needs to adjust its position and speed to maintain a fixed, short distance to its nearest neighbors, to ensure a tight flock. In addition, there may be a randomly manifested tendency for individuals to move toward the center of the flock, to ensure that all birds experience approximately the same risk of predation. Birds flying through a cluttered environment, on the other hand, are likely to be in a different behavioral state because this situation poses a different set of challenges.

### A mathematical model of aperture choice

Here we present a simple mathematical model that captures the behavior of the birds when they are confronted with the task of choosing between two apertures, and incorporates the factors and tradeoffs that could influence the passage of a flock of budgerigars through the two apertures.

We assume that the width of the left-hand aperture is *d*, and that of the right-hand aperture is (*D-d*), where *D* is the total width of the two apertures. When 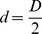, the two apertures are of equal width. We assume that the time T taken for a single bird to fly though a passage is inversely proportional to the width of the passage. While we do not know if this assumption is exactly true, it is a reasonable first approximation, given that (a) the narrower the passage, the greater the difficulty in negotiating it, and the longer the bird will take to pass through it; and (b) if visually guided flight dynamics of budgerigars are similar to bees, the speed of their flight through a passage will be proportional to the width of the passage [18], so that the time required to fly through the passage will be inversely proportional to its width. Indeed, there is recent evidence that budgerigars use optic-flow cues to regulate their flight speed, which would lead to this kind of behavior [19].

Thus, the times *T_L_* and *T_R_* taken by a bird to fly through the left- and right-hand apertures will be given respectively by
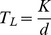
(2)

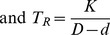
(3)where K is a constant of proportionality.

When the two apertures are of equal width, we have 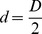, which leads to
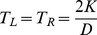
(4)If a flock of *N* budgerigars encounters the two apertures, and if *N_L_* of them choose to fly through the left-hand aperture and *N_R_* through the right-hand aperture (*N_L_*+*N_R_* = *N*), the time required for the *N_L_* birds to transit the left-hand aperture will be, from (2),
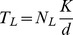
(5)and the time required for the *N_R_* birds to transit the right-hand aperture will be, from (3),
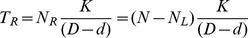
(6)Let us now consider, in turn, a number of ways in which the birds might choose between the two apertures and examine, for each case, the time taken by the entire flock to pass through the twin-aperture obstacle.

#### Strategy A: All birds choose to fly through the left-hand aperture, irrespective of its width

This situation would prevail if all of the birds had a strong left-bias.

**Figure 5:**
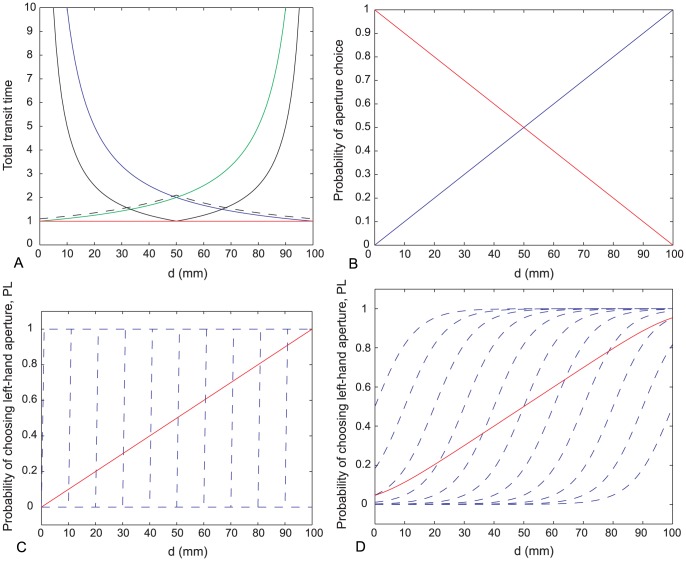
A. Illustration of total transit times as predicted by a model of a flock of budgerigars negotiating two simultaneously presented apertures of width d mm (left-hand aperture) and (D-d) mm (right-hand aperture), where D, the sum of the widths of the two apertures, is 100 mm. The curves show the variation of the total transit time with d for strategies A (blue), B (green), C (black), D (dashed black) and E (red), as described in the text. For clarity, the curve for strategy D is shown displaced slightly upwards. **B**. Probability functions for the choice of the left-hand aperture (blue curve) and the right-hand aperture (red curve) as a function of the width d of the left-hand aperture, for the optimum strategy (E) described in the text. **C**. Model showing choice probability for the left-hand aperture as a function of its width, for individual birds with a range of different bias parameters (B) varying from 0 mm to 100 mm in steps of 10 mm. The choice probability for each bird is modeled by a step function (dashed blue curve). The continuous red curve shows the resulting average choice probability function for the entire flock. **D**. Choice probability functions for the left-hand aperture for individual birds with a range of different bias parameters (B) varying from 0 mm to 100 mm in steps of 10 mm. The choice probability for each bird is modeled by a logistic function (dashed blue curve). The continuous red curve shows the resulting average choice probability function for the entire flock.

In this case, the total transit time *T_T_* taken by the entire flock will be (from equation (5)):
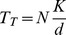
(7)
Fig. 5*A* shows how the total transit time *T_T_* for this strategy will vary as a function of the width *d* of the left-hand aperture (blue curve). The total transit time is expressed as a multiple of Kd, i.e. we assume Kd = 1.0. This is an arbitrary assignment that scales all the curves by the same factor, and does not sacrifice any generality. Always choosing to fly through the left-hand aperture is unlikely to be an efficient strategy, because the right-hand aperture is never used by any bird.

#### Strategy B: All birds choose to fly through the right-hand aperture, irrespective of its width

This situation would prevail if all of the birds had a strong right-bias.

In this case, the total transit time *T_T_* for the entire flock will be (from equation (5)):
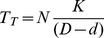
(8)
Fig. 5*A* shows how the total transit time *T_T_* for this strategy will vary as a function of the width *d* of the left-hand aperture (green curve). Always choosing to fly through the right-hand aperture is unlikely to be an efficient strategy, because the left-hand aperture is never used by any bird.

#### Strategy C: Birds choose randomly between the two apertures, irrespective of their size

This strategy would prevail either if (a) each bird were to choose randomly between the two apertures, or (b) half the flock of birds had a strong left-bias and the other half a strong right-bias.

If the size of the flock is *N*, each aperture would be chosen by (*N*/2) birds, on average.

The transit time for the birds taking the left-hand aperture would be (from equation (2)):
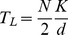
(9)and the transit timr for the birds taking the right-hand aperture would be (from equation (3)):
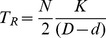
(10)The transit time *T_T_* for the entire flock to pass through the twin-aperture obstacle would be the greater of the two transit times, *T_L_* and *T_R_*. If 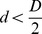, then it is clear that *T_L_* will be greater than *T_R_*; and if 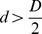, the opposite will be true. Therefore, the total transit time *T_T_* for this strategy will be:
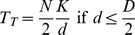
(11)i.e. if the right-hand aperture is wider than the left-hand one, and
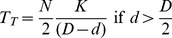
(12)i.e. if the left-hand aperture is wider than the right-hand one.

Fig. 5*A* shows how the total transit time *T_T_* for this strategy will vary as a function of the width *d* of the left-hand aperture (continuous black curve). This strategy is not necessarily optimal, because it chooses wide apertures just as frequently as it does narrow apertures.

#### Strategy D: Birds always choose the larger of the two apertures

If the left-hand aperture is wider, i.e., if 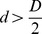, the total transit time will be
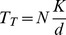
(13)If the right-hand aperture is wider, i.e., if 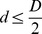, the total transit time will be
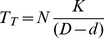
(14)
Fig. 5*A* shows how the total transit time *T_T_* for this strategy varies as a function of the width *d* of the left-hand aperture (dashed black curve). This strategy is not necessarily optimal, because the narrower aperture is never used by any bird.

#### Strategy E: Birds choose the two apertures with probabilities proportional to their relative widths

In this scenario the aperture of width *d* is chosen with probability 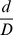, and the aperture of width (*D-d* ) is chosen with probability 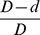. If the size of the flock is *N* (where N is a large number) then, on average, 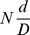 birds would choose the aperture of width *d*, and 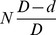 birds would choose the aperture of width (*D-d* ). The transit time for the aperture of width *d* would then be 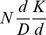, or 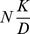. The transit time for the aperture of width (*D-d* ) would be 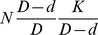, which is also equal to 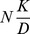. We note that, with this strategy, (a) the transit times are the same for both apertures, which means that both groups of birds will finish flying through their respective apertures at the same time; and (b) the transit times are independent of the relative widths of the two apertures. This is because the load (the number of birds) at each aperture is matched to the speed at which the birds can fly through that aperture. Since both apertures become clear at the same time, neither aperture is under-utilized, and this is the most efficient way to route traffic through the two apertures. The total transit time *T_T_* for this strategy is 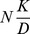, and is shown by the red curve in Fig. 5*A*. This represents the best (lowest) total transit time among all of the strategies. Importantly, in this case the transit time is not only minimal, but is independent of the relative widths of the two apertures.

The optimum strategy for minimizing the overall transit time, therefore, is to ensure that the *probability of choosing each aperture is proportional to the width of that aperture*. This leads to the optimum choice probability function shown in Fig. 5*B*. The probability of choosing the left-hand aperture is 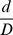, and the probability of choosing the right-hand aperture is 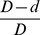 where *d* is the width of the left-hand aperture and *D-d* is the width of the right-hand aperture.

Are the budgerigars indeed realizing this optimal strategy? To investigate this, we can begin by modeling each bird's choice behavior by a unit step function, as a simple first approximation. This step function is described by *u*(*d-B*), where *u*, the probability of choosing the left-hand aperture when it has a width *d*, is equal to 0 when d≤B, and 1 when *d*>B. B is a parameter that represents the bird's bias. The bird is unbiased if B = 50 mm, left-biased if B<50 mm, and right-biased if B>50 mm. A family of choice probability functions, for birds with different biases, is shown in Fig. 5*C*.

The desired optimum choice probability function for the entire flock can be realized by having a different bias parameter for each bird. If B varies uniformly over the range [0,D], it can be shown that the choice probability function for choosing the left-hand aperture for the entire flock will be 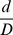, as illustrated by the continuous red curve in Fig. 5C.

The proof of this is as follows:

The probability of choosing the left-hand aperture, averaged over a large number of birds with biases distributed uniformly over the range [0,D], is given by the expected value of the step function *u(d-B)*. Denoting this expected value by *P_L_*, we have
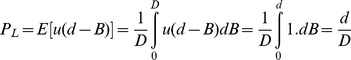
(15)The average probability of choosing the right-hand aperture, *P_R_*, is given by 1-*P_L_*, which is 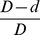. These functions are exactly those illustrated in Fig. 5B. Therefore, the optimum strategy illustrated in Fig. 5*B* can be realized by a flock of birds in which the individual biases are distributed uniformly over the range [0,D].

In reality, we see that the choice probability curves for the individual birds are not exactly step functions. Rather, they are approximately sigmoidal in shape, as is evident from the data in Fig. 3. Let us approximate the probability that a particular bird will choose the left-hand aperture by the logistic function
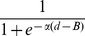
(16)where *d* is the width of this aperture, B is the bias parameter (as before), and α is a parameter which defines the sharpness of the bird's transition between the left-hand aperture and the right-hand one. The larger the value of α, the steeper the transition; when α = ∞, we have a step function, as above.

The dashed curves in Fig. 5*D* show choice probability functions for the left-hand aperture, modeled according to the logistic function, with α = 0.15, for birds with various bias parameters (B) ranging from 0 mm to 100 mm in steps of 10 mm.

Proceeding as before, we can calculate the probability of choosing the left-hand aperture, averaged over a large number of birds with biases distributed uniformly over the range [0, D]. This is done by evaluating the expected value of the logistic function 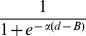. Denoting this expected value by *P_L_*, we have
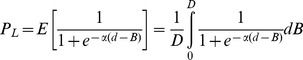
(17)The integral in (17) can be evaluated by setting
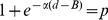
(18)which leads to
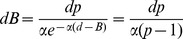
(19)Thus, we have
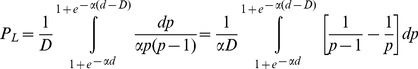
(20)i.e.
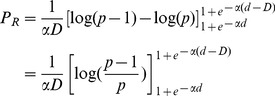
(21)which gives
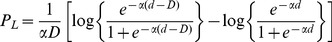
(22)This can be simplified to read
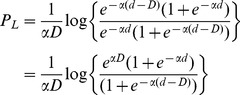
(23)
*P_L_* is the probability of choosing the left-hand aperture as a function of its width (*d*). It is plotted as the continuous red curve in Fig. 5*D*. We see that this function is very similar in shape to the optimal choice probability function for strategy E, illustrated by the red curve in Fig. 5*C*.

Therefore, we can say that the desired optimal strategy can be approximated well by a flock of birds in which the choice probability function for each bird is characterized by a sigmoidal function, and where the biases of the various birds vary over a wide range, going from early extreme left, through zero, to nearly extreme right. The data from the birds that we have tested suggest that this is indeed what occurs.

While the optimal strategy (for the flock as a whole) is one in which each aperture is chosen with a probability that is proportional to its width, in practice this probability likely to be a broad sigmoidal function of aperture width, rather than a linear function. This is because apertures that are narrower than about 30 mm are not traversable. The pooled data for all the birds (Fig. 3*F*) does indeed display a broad sigmoidal choice-frequency profile with these properties.

### Aperture choice in the context of the Matching Law of choice behavior in experimental psychology

The optimum overall choice probabilities predicted by the model, as shown by the red and blue curves in Fig. 5B, are very reminiscent of the so-called “Matching Law”, which states that an animal's choices between two options tend to be proportional to the relative benefit (or reward) that is offered by each option [20], [21]. Interestingly, while individual birds display “overmatching” and varying degrees of “bias” as characterized by Equation (2) in [21], the predicted behavior of the flock as a whole follows the classic, proportional Matching Law, as postulated in the pioneering work of Herrnstein (1961).

### Inter-individual variations in the discrimination of relative gap width

Our study also finds that individual birds display different sensitivities to changes in gap width– that is, the transition from preferring the left-hand gap to the right-hand gap occurs over a smaller change in gap width for some birds, than for others. This variation in sensitivity is captured by the variations in the parameter *Δd* (Table 2). The reasons for this variation across individuals are presently unclear. They could arise simply from individual differences in sensory discrimination capacity. Alternatively, evolution may have tailored these differences to fine-tune and better optimize flock performance. For example, in a flock with a large number of birds, the optimum overall choice probability functions (as shown by the red and blue curves in Fig. 5B) could be achieved by having individuals with sharp discrimination of gap widths (small *Δd*), but with a large variety of biases. In a flock with a small number of birds, on the other hand, there can only be a few different biases, and so the optimum overall choice probability functions can be realized only if the discrimination of gap widths is relatively poor (large *Δd*). Further work, investigating individuals from different-sized flocks, would be required to investigate this possibility.

## Methods

### Ethics statement

All experiments were carried out in accordance with Australian Laws on the protection and welfare of laboratory animals and the approval of the Animal Experimentation Ethics Committees of the University of Queensland, Brisbane, Australia (Permit QBI/646/07/ARC).

### Subjects

Adult male and female wild type budgerigars (n = 5, approximately 1 year old) served as subjects for the experiments. The birds were obtained from different local breeders. They were reared in aviaries and did not have the opportunity to fly outdoors in flocks. Male budgerigars were identified by a characteristically green plumage and a distinctly blue nasal coloration while the females had a characteristic pink nasal coloration. The birds were housed in pairs in identical cages of length 470 mm, breadth 345 mm and height 820 mm, and were not under acoustic or visual isolation. All of the birds were housed indoors in a room (length 4740 mm, width 2940 mm, height 3320 mm). Indoor lighting was provided by Phillips daylight fluorescent tubes. The lights were controlled by an automatic timer (HPM, Excel Light Switch and Timer, Cat XL 770 T), which provided a 12∶12 L∶D photoperiod. Food and water were provided ad libitum. The food was commercial budgerigar seed mix (Trill, budgerigar seed mix, Wacol, Queensland, Australia) containing a mixture of seeds, shell grit and essential vitamins and minerals. The birds were also fed occasionally with apples and greens. Daily, the birds were moved to an adjoining screened patio, of length 5400 mm, width 2300 mm and height 1800 mm, where they were released from their cages and allowed to fly between perches. This enclosure provided the opportunity for regular flight as well as exposure to natural daylight. It also contained a bird bath.

### Experimental arena

The budgerigars were flown in a purpose-built climate-controlled corridor (temperature: 23–25°C, relative humidity: 35–40%) of dimensions 7280 mm (length), 2440 mm (height) and 1360 mm (width), as illustrated in Fig. 1. The walls and floor were painted with a white, low sheen acrylic paint. Each wall was decorated with vertically oriented black, machine-cut cardboard stripes, 110 mm wide and separated by 110 mm edge to edge. Illumination was provided by four lamps in the ceiling, each carrying two 36 W fluorescent tubes (L 36W/880 Osram Skywhite FLH1) driven by a 40 kHz ballast to avoid any perception of flicker.

Halfway along the tunnel (3000 mm from the start) the birds encountered a barrier, which presented two vertically oriented apertures. The barrier was composed of cloth panels, stretched from the ceiling to the floor, to prevent accidental injury to the birds. The apertures were created by using three panels to create two vertical slits (Fig. 1). Each panel was composed of a cloth that carried a black-white checkerboard texture, of check size 40 mm×40 mm, printed on it (SJCLOTH91418, Studio Jet Instant Dry 180MIC, GBC Australia.

### Training of birds

Male and female budgerigars were brought individually into one end of the corridor. The birds were induced to take off from a hand-held perch when it was slowly rotated, and were trained initially to fly to the other end of the corridor to receive a food reward. In the later stages of training the food reward was dispensed with: the birds automatically took off when the perch was rotated, flew through the aperture and continued to the other end of the corridor, where they left the tunnel through a door at the far end, to be reunited with their companions. For each bird, this shaping and training procedure required approximately 30 flights, spread over 3 days. Once training was complete, the bird was flown under different experimental conditions and filming was commenced.

### Filming of flights

Flights of individual birds were captured in three dimensions using two high-speed video cameras (DRS lightning RDT, DRS Technologies Inc, USA) at a frame rate of 250 frames/sec. The cameras were controlled by a custom configured Pentium 4 computer running special-purpose software (*Midas 2.0*, Xcitex, Inc, USA). One camera was placed at the center of the ceiling of the corridor, looking downwards. The other camera was placed at the center of the end wall of the corridor from where the birds commenced their flight, and looked horizontally along the axis of the corridor. Each flight yielded two synchronized image sequences, one representing an overhead view of the bird and the other a rear view of the bird during the flight along the corridor.

### Camera calibration

Stereo calibration of the cameras was carried out using a reference checkerboard pattern (check size 150 mm×150 mm) in association with the J.Y. Bouguett camera calibration toolbox [22], [23]. This procedure delivered the calibration parameters for each camera (including characterization of imaging distortions) and also determined the precise 3-D position and orientation of one camera with respect to the other. Tests with calibration markers indicated that the system had an absolute positional accuracy of ca. 10 mm×10 mm×10 mm.

### Digitization and reconstruction of flight trajectories

The video cameras were run at a frame rate of 250 frames per second. The image co-ordinates of the center of the head of the bird in each pair of synchronized frames were digitized interactively using a computer mouse and a custom-designed Matlab program. The head was clearly visible by virtue of the natural yellow patch that it carried (which appears white in a black and white image). The sequences of head co-ordinates obtained from the two image sequences were then used in conjunction with the camera calibration parameters to reconstruct the trajectory of the head in 3-D, through the entire flight sequence. Trajectories were plotted without down-sampling the data.

### Apertures

The apertures were presented halfway along the tunnel in a transversely oriented wall. Each aperture was oriented vertically and extended from the floor to the ceiling, as shown in Figure 1. The two apertures were created by constructing the transverse wall out of three panels. There were two outer panels, each 450 mm wide extending inwards from the side walls. In addition there was a central panel, 340 mm wide. All of the panels carried the checkerboard pattern. The relative widths of the two apertures were varied systematically, in different experiments, by changing the position of the central panel along the width of the tunnel. When the central panel was positioned exactly midway between the two outer panels, each aperture was 50 mm wide. Displacing the central panel to the left caused the left-hand aperture to become narrower and the right-hand aperture to become wider, and vice versa. By varying the position of the central panel in steps of 10 mm, the relative widths of the two apertures were varied systematically from one extreme of 0 mm (left) and 100 mm (right), through the symmetrical position of 50 mm (left) and 50 mm (right), to the other extreme of 100 mm (left) and 0 mm (right), as shown in Table 1. The 11 different experimental conditions were presented in random sequence, as prescribed by a computer-generated sequence of random numbers generated using Matlab (Mathworks, USA).

### Birds and trials

5 birds were used in the experiments: *One, Casper, Two, Drongo* and *Saras*. At the time of conducting these experiments, our capacity to hold and maintain birds in an ethically proper environment was restricted to 5 birds.

Each bird was tested on each of the experimental conditions for between 6 and 14 trials, so that each bird made a total of 106–107 choices. The data were analyzed to obtain the choice frequency (expressed as a percentage of the total number of choices) for the left-hand aperture, for each experimental condition and for each bird. Thus, if a particular bird chose the left- hand aperture in 8 out of 11 trials in one particular experimental condition, its choice frequency for the left-hand aperture was calculated as 100×(8/11)% = 73%. The choice frequency for the right-hand aperture was then 100%-73% = 27%.

### Statistical analysis of data

The choice frequencies for the apertures were analyzed to determine whether they were significantly different from the random-choice level of 50%. If a bird chooses the left-hand aperture n times out of N trials, the probability of choosing the left-hand aperture α is n/N. Assuming that the bird's choice behavior follows a binomial distribution, the standard error of the mean of this distribution, σ, can be calculated as 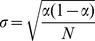 [24]. This value of σ is then used in a standard two-tailed t-test to determine whether α is significantly different from the random-choice level of 50%, as described in [24] and [25].

### Quantification of aperture discrimination

**Figure 6:**
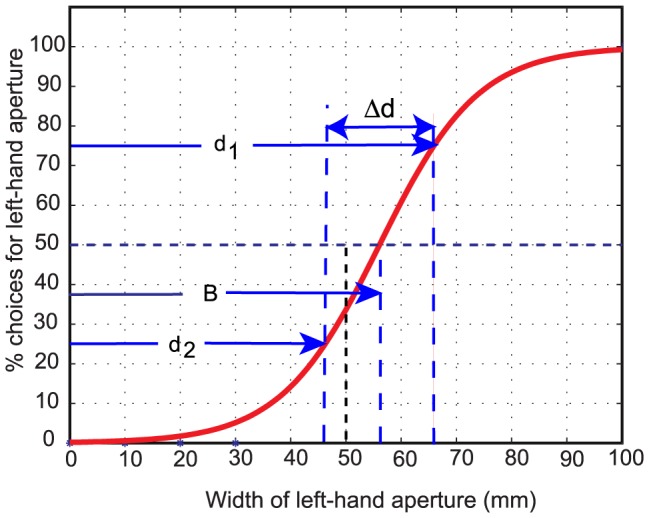
Logistic function used to quantify aperture discrimination. Details in text.

We quantified the capacity of the birds to discriminate differences in aperture width by fitting the choice frequency data to a logistic function [26] that describes the choice frequency *F_L_* for the left-hand aperture as
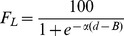
(24)where *d* is the width of the left-hand aperture. This function is illustrated in Fig. 6.

B is a bias parameter that specifies the bias of the bird, as estimated from the fitted function. α is a parameter which defines the sharpness of the bird's transition between the left-hand aperture and the right-hand one. The parameters B and α, and their 95% confidence intervals were determined by performing a least-squares fit of the logistic function to the data using the NLINFIT and CI routines of Matlab (Mathworks, USA). The logistic function was chosen to model the data because (a) it is a relatively simple function (b) it is perfectly anti-symmetrical about the 50% choice frequency level, as is required by the reciprocal relationship between the widths of the left- and right-hand apertures. However, other anti-symmetrical functions could have been used instead, and would have yielded similar results.

From the fitted logistic functions we can also estimate the sharpness of each bird's ability to discriminate changes in aperture size by calculating the change in aperture width (*Δd*) that is required for the choice frequency of the bird for the left-hand aperture to increase from 25% to 75%. This is carried out as follows. d_1_, the width of the left-hand aperture that elicits a choice frequency of 25% for this aperture, is given by the relationship
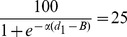
(25)from which we can solve for d_1_:
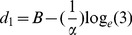
(26)Similarly d_2_, the width of the left-hand aperture that elicits a choice frequency of 25% for this aperture, is given by the relationship
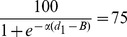
(27)from which we can solve for d_2_:
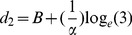
(28)Therefore *Δd*, the change in aperture width required for the preference of the left-hand aperture to increase from 25% to 75% is given by
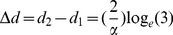
(29)We note that *Δd* is inversely proportional to the value of α. It does not depend upon the parameter *B*, which specifies the bias of the bird. This is appropriate, because *Δd* is meant to indicate the sharpness of the transition of the bird's preference from the one aperture to the other, irrespective of where this transition occurs.

### Note added in proof

A recent study [27], published while this paper was under review, has demonstrated that budgerigars also display individually varying lateralization in other tasks such as choice of landing location, or choice of foot used to climb on to a perch.

## Supporting Information

Video S1 Example of a flight in which a bird (Casper) selects the left-hand aperture (of width 60 mm) over the right-hand aperture (of width 40 mm).
(ZIP)
